# The Relationship Between Cytokine Regulation and Anti-Inflammatory Action of Amine-Carboxyborane in L929 Fibroblasts and IC-21 Macrophages

**DOI:** 10.1155/MBD.1995.257

**Published:** 1995

**Authors:** Margaret E. Murphy, Robert P. Shrewsbury, Anup Sood, Bernard F. Spielvogel, Amy L. Elkins, Iris Hall

**Affiliations:** 1 Division of Pharmaceutics School of Pharmacy University of North Carolina Chapel Hill North Carolina 27559-7360 USA; 2 Division of Medicinal Chemistry & Natural Products School of Pharmacy University of North Carolina Chapel Hill North Carolina 27559-7360 USA; 3 Boron Biologicals, Inc. 620 Hutton St. Raleigh N.C. 27606 USA

## Abstract

The amine-carboxyboranes anti-inflammatory agents were shown to block TNF α release at 90 min. and IL-1 release at 5 hr. from macrophages. The agenst competed with L929 fibroblasts high affinity receptors for endogenous cytokines which regulate the inflammation process. Blocking the TNFα receptor at 90 min. by the agents from 10 to 50  μΜ, resulted in lysosomal hydrolytic enzyme inhibition and lowering of prostaglandin synthesis as well as reductions in calcitonin high affinity receptor binding and calcium influx into the cells. IL-1 receptors when blocked by the agents at 5 hr. resulted in a reduction of NAG activity and leukotriene synthesis. An elevation of proline incorporation into collagen occurred at 90 min. and 24 hr. in the presence of the agents.


### THE RELATIONSHIP BETWEEN CYTOKINE REGULATION AND ANTI-INFLAMMATORY ACTION OF AMINE-CARBOXYBORANE IN L929 FIBROBLASTS AND IC-21 MACROPHAGES

Margaret E. Murphy[1], Robert P. Shrewsbury[1], Anup Sood[3], Bernard F. Spielvogel[3], Amy L. Elkins[2] and Iris Hall[2]

[1] Divisions of Pharmaceutics and
[2] Medicinal Chemistry & Natural Products
School of Pharmacy, University of North Carolina Chapel Hill, North Carolina 27559-7360, U.S.A.
[3] Boron Biologicals, Inc., 620 Hutton St., Raleigh, N.C. 27606, U.S.A.

**Abstract**

The amine-carboxyboranes anti-inflammatory agents were shown to block TNFα release at 90 min. and IL-1 release at 5 hr. from macrophages. The agenst competed with L929 fibroblasts high affinity receptors for endogenous cytokines which regulate the inflammation process. Blocking the TNFα receptor at 90 min. by the agents from 10 to 50 μM, resulted in lysosomal hydrolytic enzyme inhibition and lowering of prostaglandin synthesis as well as reductions in calcitonin high affinity receptor binding and calcium influx into the cells. IL-1 receptors when blocked by the agents at 5 hr. resulted in a reduction of NAG activity and leukotriene synthesis. An elevation of proline incorporation into collagen occurred at 90 min. and 24 hr. in the presence of the agents.

**Introduction**

Amine-carboxyboranes and related derivatives have been shown to be potent anti-inflammatory and anti-osteoporosis agents in rodents[1-4]. These agents were shown to block septic shock, pleurisy, arthritis and induced edema in mice at doses from 2 to 8 mg/kg[1]. Previous studies have demonstrated that these agents reduced hydrolytic enzyme activities, in addition to prostaglandin and leukotriene synthesis in macrophages, leukocytes and Be Sal osteoporotic cells[3]. Other studies have indicated <u>in vivo</u> and <u>in vitro</u> that the amine-carboxyboranes reduced TNFα and IL-1 levels after challenge with LPS[1,2] . Cytokine release from inflammatory cells is related to the induction of fever, acute phase response and release of secondary chemical mediators, e.g. prostaglandins and leukotrienes[5]. IL-1 is a pyrogenic and chemotaxic factor[6]. Studies have demonstrated that TNFα is maximally released from IC-21 mouse macrophages from 60 to 90 min whereas IL-1 is maximally released from $P_{388D}$ macrophages at 5 to 8 hr[1].

**Methods**

The amine carboxyborane derivatives were previously synthesized and the chemical and physical characteristics reported [1]: Compound #<u>1</u> $(C_6H_5)_3PBH_2COOH$, Compound #<u>2</u> $C_{16}H_{33}N(CH_3)_2BH_2COOH$ and Compound #<u>3</u> $(CH_3)_3NBH_2COOCH_3$. The standards, Tumor necrosis factor alpha, TNFα and interleukine-1, IL-1, were obtained from Sigma Chemical Co. (St. Louis, MO).

Cell Maintenance[1,7]]
IC-21 mouse macrophages (RPMI 1640 + 10% FCS + P/S) and L929 mouse fibroblasts (EMEM + 10% FCS + P/S) were grown to confluency in 6 well plates for the following assays. IC-21 macrophages were selected because they release cytokine as well as hydrolytic enzymes and secondary chemical mediators of inflammation. L929 fibroblasts were selected because they are inflammatory target tissue cells which contain high affinity receptors for cytokines and are responsible for wound repair after injury has occurred. TNFα release from IC-21 macrophages peaks at 90 min. and the effects of the agents from 12.5 to 50 μM were determined at 90 min and 5 hr. IL-1 release from $P388_D$ macrophages was determined at 90 min. and 5 hr. The cytotoxic L929 bioassay was used to quantitate the cytokine levels[1,7].

High Affinity Binding to Receptors on L929 or IC-21 Cells[8]
Two μCi of $^{125}$I-TNFα (human recombinant , 30 mCi/ug New England Nuclear) or $^{125}$I-IL-1 (70-120μCi/μg, New England Nuclear) was added to confluent L929 or IC-21 mouse fibroblasts or macrophages for 30 to 120 min with the amine-carboxyboranes from 10 to 50 μM. These cells were chosen because they have plama membrane high affinity receptors for these specific cytokines. After the allotted time period, the medium was gently decanted and the cells were washed 6 X in cold isotonic PBS, pH 7.2. The cells were taken up in 0.1N NaOH and aliquots were counted. $^{125}$I-Calcitonin (human, 2000 Ci/nmole, Amersham) or $^{3}$H-1,25-dihyroxyvitamin $D_3$ (155-175 Ci/mmole, New England Nuclear) [10 μCi] was added to confluent L929 cells with drugs from 10 to 100 μM and incubated 90 min or 5 hr. Cells were washed repeatedly in PBS and taken up in 0.1 N NaOH. In the case of 1,25-dihydro-vitamin $D_3$, the nuclei preparation was obtained. The aliquots were counted in a Pachard beta counter corrected for quenching, and non-specific binding of isotopes.

$^{45}$Ca Uptake by Cells and $^{3}$H Proline Incorporation into Collagen of L929 Cells[9,10]
$^{45}CaCl_2$ (0.2 mCi, New England Nuclear) was added to confluent L929 cells. The medium was decanted and the cells were washed 4 times with PBS, pH 7.2. The cells were taken up in 0.1N NaOH and counted. L929 cells (70% confluent) were incubated with 1 μCi of 2,3,4,5-$^{3}$H-proline (102 Ci/mmol, New England Nuclear) and drugs. After 24 hr, the medium was discarded and the cells were washed repeatedly with PBS. The cells were harvested in 0.1N NaOH. After treatment with 20% TCA, cells were centrifuged at 3500 g X 5 min. The supernatant was discarded and the pellet was suspended in 1 ml of 50 mM Tris + 5 mM $CaCl_2$ + 20 μl of collagenase (10 mg/ml buffer, [Sigma Chemical Co. St. Louis, MO]) and incubated for 2 hr at 37°C. Tannic acid:TCA [1:1](5%) solution was added and incubated for 15 min at room temperature followed by centrifugation to separate the collagen from non-collagen protein[11].

Lysosomal Enzymes
IC-21 macrophages [$10^8$] were grown to confluency and incubated for 90 min or 5 hr. with drugs at 12.5, 50 and 100 μM . Acid phosphatase activities were determined using 0.1 M β-glycerolphosphate in 0.1 M acetate buffer, pH 5.0 [1-3]. Total enzyme was released with Triton X-100. The reaction was terminated with 10% TCA and then the solution was centrifuged at 3500 g for 10 min. The inorganic phosphate in the supernatant was determined spectrophotometrically at 800 nm by the method of Chen et al.[12]. N-Acetylglucosaminidase [NAG] activity was determined by the method Tulberg-Reinert and Hetti[13]. IC-21 macrophages were incubated with drugs at 12.5, 50 and 100 μM for 90 min, and 5 hr in the presence of 10 ug/ml LPS (Salmonella abortus equi). The medium [50 μl] was incubated with 7.5 mM *p*-nitrophenyl-N-acetyl-β-D-glucosaminide in glycine buffer, pH 5.0 in 96-well microplates. The reaction was stopped by the addition of glycine/EDTA buffer [50mM:5mM]. The concentration of *p*-nitrophenol released was measured at 405 nm using a microplate reader [Thermomax, Molecular Devices Corp.].

Prostaglandin Synthetase Activity

The incubation procedures of Tomlinson et al.[14] and Glatt et al.[15] were used to determine prostaglandin formation from $^3$H(N)-arachidonic acid (100 Ci/mmole) and IC-21 cells ($10^6$). After 1 hr, the reaction was terminated with 2N HCl and the mixture was extracted with ether and evaporated. The residue was dissolved in ethyl acetate and applied to silica gel TLC plates which were eluted with chloroform, methanol, water and acetic acid (90:8:1:0.8). The plates were developed with iodine vapor and the area appropriate to the prostaglandin standards was scraped and counted [14,15]. The dpm in each area was calculated as percent of the total dpm applied to the plate.

5'-Lipoxygenase Assay

IC-21 cells were incubated for 30 min with phosphate buffer (pH 7.2) containing 0.6 mM $CaCl_2$, and 1.0 mM $MgCl_2$, 10 mg Calcium Ionophore A23187 and 1 mCi $^{14}$C-arachidonic acid (100 Ci/mmol). The reaction was terminated with 2 volumes of EtOAc:$CH_2Cl_2$ containing 12 mg cold arachidonic acid. The organic phase was evaporated to a residue which was applied to silica gel plates. The plates were eluted with chloroform:methanol:water:acetic acid (90:8:1:0.8). The 5-HETE area corresponding to the standard was scraped and counted [16,17].

**Results**

The LPS induced TNFα release from IC-21 cells and IL-1 release from $P388_{D1}$ cells was maximum at 90 min. and 5 hr., respectively. LPS concentration of 5, 10 and 20 μg/ml of growth medium were examined in both assays and 10 μg/ml afforded the best release of TNFα at 90 min and IL-1 at 5 hr. Thus, this concentration of LPS was selected for the following studies. The reduction of TNFα release caused by amine-carboxyboranes from 12.5 to 50 μM followed a concentration-response curve over at 90 min. and 5 hr [Table 1]. It was interesting to note that the agents still suppressed TNFα release from IC-21 macrophages at 5 hr. long after the peak release of the cytokine induced by LPS.

The effect of amine-carboxyboranes on the release of IL-1 from $P388_{D1}$ cells using 10 μg/ml LPS at 90 minutes and 5 hours was reduced at all concentrations of agents, but most significantly at 25 and 50 μM (43-100%) [Table 2]. Furthermore, after 90 minutes in the presence of 50 μM of Compounds #1 and #3, and after 5 hours with 50 μM of Compound #3, IL-1 release was inhibited 100%. The agents were able to suppress IL-1 release from $P388_{D1}$ cells at an early time i.e 90 min., when IL-1 levels were normally substantially lower after LPS induction [Table 3].

**Table 1. The Effect of Amine-Carboxyboranes on TNFα Release from IC-21 Cells in the Presence of 10 μg/ml LPS**

% of Control [N=8]

| Conc. μM | COMPOUND #1 90 min | COMPOUND #1 5 hrs | COMPOUND #2 90 min | COMPOUND #2 5 hrs | COMPOUND #3 90 min | COMPOUND #3 5 hrs |
|---|---|---|---|---|---|---|
| 0 | 100±5[a] | 100±4[b] | 100±5[a] | 100±4[b] | 100±5[a] | 100±4[b] |
| 12.5 | 81±4 | 95±4 | 90±6 | 86±5 | 50±5* | 97±6 |
| 25 | 60±4* | 66±5* | 55±4* | 52±4* | 54±4* | 57±5* |
| 50 | 41±5* | 29±3* | 31±5* | 31±3* | 33±4* | 29±3* |

[a]Control=171 ng TNFα released; [b]Control=154.2 ng TNFα released; *p< 0.001

---

**Table 2. The Effect of Amine-Carboxyboranes on IL-1 Release from $P388_{D1}$ Cells in the Presence of 10 μg/ml LPS**

% of Control [N =8]

| Conc. μM | COMPOUND #1 90 min | COMPOUND #1 5 hrs | COMPOUND #2 90 min | COMPOUND #2 5 hrs | COMPOUND #3 90 min | COMPOUND #3 5 hrs |
|---|---|---|---|---|---|---|
| 0 | 100±6 [a] | 100±6 [b] | 100±6 [a] | 100±6 [b] | 100±6 [a] | 100±6 [b] |
| 12.5 | 98±7 | 96±6 | 93±5 | 57±4* | 96±6 | 91±5 |
| 25 | 32±3* | 51±4* | 31±3* | 52±6* | 31±2* | 32±3* |
| 50 | 0 | 52±6* | 57±5* | 52±4* | 0 | 0 |

[a]Control=91.2 ng IL-1 released; [b]Control=166.8 ng IL-1 released; *p <0.001

---

TNFα high affinity receptor binding on L929 cells was reduced with amine-carboxyboranes at 10 μM from 30 to 120 min. This is the same period of time when the maximum release of TNFα occurs from IC-21 macrophages. Higher concentration of the agents after 90 min incubation did not improve the inhibition of TNFα high affinity binding to L929 fibroblast high affinity receptors [Table 4].

---

**Table 3 The Effect of Amine-Carboxyboranes at 10 μM on TNFα High Affinity Receptor Binding on L929 Cells**

% of Control [N =6]

| Time (min.) | Control | COMPOUND #1 | COMPOUND #2 | COMPOUND #3 |
|---|---|---|---|---|
| 30 | 100±16[a] | 75±9 | 74±7* | 66±14* |
| 60 | 100±13[b] | 84±6 | 78±12** | 82±7 |
| 90 | 100±12[c] | 89±8 | 62±8* | 44±5* |
| 120 | 100±9[d] | 12±5* | 63±7* | 17±5* |

Control Values: [a] 3656 CPM/mg protein; [b] 6208 CPM/mg protein; [c] 12819 CPM/mg protein; [d] 11276 CPM/mg protein; *p<0.0001; **p<0.005.

---

IL-1 high affinity binding to L929 receptors was determined after 1.5, 5, 8, 24, 30 and 48 hours in the presence of amine-carboxyboranes at 10 μM [Table 5]. IL-1 high affinity receptor binding to L929 cells significantly decreased over time by 53-68% after 5 hours for Compounds # 2 and #3; however Compound #1 caused only a 13% decrease in binding at 90 minutes. IL-1 high affinity receptor binding then increased again in the presence of derivatives from 8 hours to 48 hours, At this latter time, i.e. 48 hr., IL-1 binding reached levels statistically above control values.

**Table 4. The Effect of Amine-Carboxyboranes on TNFα High Affinity Receptor Binding on L929 Cells After 90 Minutes**

% of Control [N =6]

| Conc (μM) | COMPOUND #1 | COMPOUND #2 | COMPOUND #3 |
|---|---|---|---|
| 12.5 | 83±5 | 76±4* | 100±9 |
| 25 | 78±4* | 94±5 | 109±6 |
| 50 | 95±7 | 110±10 | 121±14 |

Control = 100±14% (1435 CPM/mg protein)* p<0.05

---

**Table 5. The Effect of Amine-Carboxyboranes at 10 μM on IL-1 High Affinity Receptor Binding on L929 Cells**

% of Control [N=6]

| Time (Hours) | Control | Compound #1 | Compound #2 | Comp ound #3 |
|---|---|---|---|---|
| 1.5 | 100±2[a] | 87±4 | 53±2* | 125±10 |
| 5 | 100±8[b] | 101±14 | 47±9* | 32±13* |
| 8 | 100±6[c] | 96±5 | 98±2 | 82±4 |
| 24 | 100±7[d] | 101±5 | 92±11 | 83±5 |
| 30 | 100±5[e] | 92±3 | 86±5 | 78±5* |
| 48 | 100±9[f] | 130±4 | 163±7c | 161±6* |

Control Values: [a] 468 CPM/mg protein; [b] 2108 CPM/mg protein; [c] 676 CPM/mg protein; [d] 439 CPM/mg protein; [e] 397 CPM/mg protein; [f] 115 CPM/mg protein; * p = 0.05.

---

IL-1 high affinity binding L929 receptors after 5 hours was significantly decreased from 41 to 55% of the control value [Table 6] at all higher concentrations employed. Amine-carboxyboranes suppress IL-1 high affinity binding to fibroblast receptors at a time where maximum IL-1 binding has been demonstrated in this cell line, i.e. 5 hr.
The amine-carboxyboranes at 12.5, 25 and 50 μM significantly decreased IL-1 high affinity binding of IC-21 receptors at 90 min. at a time when time when maximum binding occurs in these fibroblasts [Table 7]. At 12.5 μM the maximum reduction of IL-1 high affinity binding was

---

**Table 6. The Effect of Amine-Carboxyboranes on IL-1 High Affinity Receptor Binding on L929 Cells After 5 Hours**

% of Control [N =6]

| Concentration (μM) | COMPOUND #1 | COMPOUND #2 | COMPOUND #3 |
|---|---|---|---|
| 12.5 | 44±6* | 55±6* | 42±5* |
| 25 | 45±7* | 50±7* | 41±6* |
| 50 | 49±8* | 43±6* | 50±6* |

Control = 100±9%(597 CPM/mg protein);* p<0.0001

---

afforded; Compound #1 reduced binding of IL-1 by 27%, Compound #2 by 58% and Compound #3 by 53%. Increased concentrations of the derivatives did not improve the reduction of IL-1 binding to its high affinity receptor in macrophages.

---

**Table 7. The Effect of Amine-Carboxyboranes on IL-1 High Affinity Receptor Binding on IC-21 Cells After 90 Minutes**

% of Control [N=6]

| Concentration (μM) | COMPOUND #1 | COMPOUND #2 | COMPOUND #3 |
|---|---|---|---|
| 12.5 | 73±7* | 42±2* | 47±6* |
| 25 | 69±4* | 49±6* | 48±4* |
| 50 | 67±5* | 46±2* | 55±2* |

Control=100±6% (820 CPM/mg protein.); *p < 0.001.

---

There was essentially no effect of amine-carboxyboranes at 12.5, 25 and 50 μM on NAG activity in IC-21 macrophages at 90 minutes or 5 hours [Tables 8 and 9].

---

**Table 8. The Effect of Amine-Carboxyboranes on NAG Activity of IC-21 Cells After 90 Minutes**

% of Control[N=8]

| Concentration (μM) | COMPOUND #1 | COMPOUND #2 | COMPOUND #3 |
|---|---|---|---|
| 12.5 | 95±6 | 97±5 | 94±5 |
| 25 | 99±1 | 92±5 | 92±11 |
| 50 | 96±5 | 92±4 | 92±8 |

Control = 100±4% (69 nmoles *p*-nitrophenol released/ml medium)

---

**Table 9. The Effect of Amine-Carboxyboranes on NAG Activity in IC-21 Cells After 5 Hours**

% of Control [N=8]

| Concentration (μM) | COMPOUND #1 | COMPOUND #2 | COMPOUND #3 |
|---|---|---|---|
| 12.5 | 90±5 | 97±5 | 91±6 |
| 5 | 83±7 | 101±6 | 91±9 |
| 50 | 88±6 | 86±7 | 88±6 |

Control = 100±7% (80.4 nmoles *p*-nitrophenol released/ml medium)

---

IC-21 cells when incubated for 24, 48 and 72 hours in the presence of amine-carboxyboranes at 1 or 10 μM and NAG activity showed moderate reductions in NAG activity (Table 10). Compounds #1 and #3 demonstrated similar reductions in activity at 24 hours. Maximum reduction of NAG activity occurred at 48 hours at 10 μM for all compounds.

In IC-21 cells after incubation with amine-carboxyboranes for 90 minutes, acid phosphatase activity was reduced by Compound #2 at 12.5 μM and Compound #3 at 25 μM. Compound #1 caused a 74-78% reduction of acid phosphatase activity at 25 and 50 μM (Table 11). No concentration dependent pattern was evident for the three compounds at 90 min.

At 5 and 18 hours the effects of the amine-carboxyboranes on IC-21 macrophage acid phosphatase activity were generally less; nevertheless, the values were significantly reduced from the control value (Table 12-13).

**Table 10. The Effect of Amine-Carboxyboranes at 1 or 10 μM on NAG Activity in IC-21 Cells**

% of Control [N=8]

| Time (hours) | Control | Compound #1 | | Compound #2 | | Compound #3 | |
|---|---|---|---|---|---|---|---|
| | | 1μM | 10μM | 1uM | 10μM | 1μM | 10μM |
| 24 | 100±2[a] | 74±4* | 71±1* | 99±16 | 104±3 | 79±8* | 74±2* |
| 48 | 100±4[b] | 82±4* | 63±5* | 89±4 | 48±2* | 83±4 | 66+1* |
| 72 | 100±5[c] | 122±4 | 88±2 | 97±3 | 86±3 | 99±3 | 79±3* |

Control Values: [a] 84 nmoles p-nitrophenol released/ml medium; [b] 150 nmoles *p*-nitrophenol released/ml medium; [c] 174 nmoles *p*-nitrophenol released/ml medium;* p<0.0001;

---

**Table 11. The Effect of Amine-Carboxyboranes on Acid Phosphatase Activity in IC-21 Cells After 90 Minutes**

% of Control [N=10]

| Concentration (μM) | COMPOUND #1 | COMPOUND #2 | COMPOUND #3 |
|---|---|---|---|
| 12.5 | ND | 11±2* | 66±7* |
| 25 | 22±4* | 32±7* | 11±3* |
| 50 | 26±4* | 24±7* | 35±5* |

Control = 100±6% (260 mg $P_i$/mg protein/90 minutes);* p<0.0001; ND = not determined.

---

**Table 12. The Effect of Amine-Carboxyboranes on Acid Phosphatase Activity in IC-21 Cells After 5 Hours**

% of Control [N=10]

| Concentration (μM) | COMPOUND #1 | COMPOUND #2 | COMPOUND #3 |
|---|---|---|---|
| 12.5 | 84±8 | 80±7* | 93±6 |
| 25 | 78±4* | 55±2* | 58±7* |
| 50 | 25±6* | 55±8* | 51±7* |

Control = 100±7% (380 mg $P_i$/mg protein/5 hours);*p<0.0001.

---

Prostaglandin cyclo-oxygenase activity in IC-21 cells was measured in the presence of amine-carboxyboranes at 12.5, 25 and 50 μM after 90 minutes and 5 hours. Prostaglandin cyclo-oxygenase activity was reduced at all times in IC-21 macrophages. In IC-21 cells, prostaglandin cyclo-oxygenase activity was maximally reduced by Compound #1 at 90 minutes ( Table 14) and 5 hours at 50 μM (Table 15). Compounds #2 and #3 at 50 μM demonstrated better reduction of activity at 90 minutes.

---

**Table 13. The Effect of Amine-Carboxyboranes on Acid Phosphatase Activity in IC-21 Cells After 18 Hours**

% of Control ([N=10]

| Concentration (μM) | COMPOUND #1 | COMPOUND #2 | COMPOUND #3 |
|---|---|---|---|
| 12.5 | 55±5* | 63±6* | 99±7 |
| 25 | 58±6* | 55±7* | 28±3* |
| 50 | 58±5* | 62±5* | 35±6* |

Control = 100±6% (500 mg $P_i$/mg protein/18 hours);* p<0.0001

---

**Table 14. The Effect of Amine-Carboxyboranes on Prostaglandin Cyclo-oxygenase Activity in IC-21 Cells After 90 Minutes**

| | % of Control [N=6] | | |
|---|---|---|---|
| Concentration (μM) | COMPOUND #1 | COMPOUND #2 | COMPOUND #3 |
| 12.5 | 53±4* | 95±6 | 99±8 |
| 25 | 38±6* | 78±7* | 83±7 |
| 50 | 42±3* | 66±5* | 53±±6* |

Control = 100±7% (16,020 DPM/mg protein);* p<0.0001

---

**Table 15. The Effect of Amine-Carboxyboranes on Prostaglandin Cyclo-oxygenase Activity in IC-21 Cells After 5 Hours**

| | % of Control [N=6] | | |
|---|---|---|---|
| Concentration (μM) | COMPOUND #1 | COMPOUND #2 | COMPOUND #3 |
| 12.5 | 75±3* | 86±5* | 77±5* |
| 25 | 46±4* | 109±7 | 68±4* |
| 50 | 42±3* | 94±4 | 68±4* |

Control = 100±6% (14,656 DPM/mg protein);*p<0.0001.

---

5'-Lipoxygenase activity in IC-21 cells was measured in the presence of amine-carboxyboranes at 12.5, 25 and 50 μM hours after 90 minutes or 5 hours. At 90 minutes incubation the magnitude of reduction of 5'-lipoxygenase activity in IC-21 cells was less with Compounds #2 and #3 which achieved a 33-39% reduction at 50 μM (Table 16) than at 5 hours incubation, when the 5'-lipoxygenase activity was reduced 46-52% by all three of the amine-carboxyboarnes [Table 17].

---

**Table 16. The Effect of Amine-Carboxyboranes on 5'-Lipoxygenase Activity in IC-21 Cells After 90 Minutes**

| | % of Control [N=6] | | |
|---|---|---|---|
| Concentration (μM) | COMPOUND #1 | COMPOUND #2 | COMPOUND #3 |
| 12.5 | 94±9 | 70±7* | 74±8* |
| 25 | 89±5 | 69±9* | 65±6* |
| 50 | 88±7 | 67±6* | 61±7* |

Control = 100±13 % (2902 DPM/mg protein);*p<0.0001

---

The effect of amine-carboxyborane at 10 μM on calcium uptake into L929 cells was measured at 1.5, 5, 8, 24 and 48 hours (Table 18). At all times, the calcium uptake was decreased significantly and at 5 hours the uptake of calcium by L929 cells to reach its lowest level for each compound.

---

**Table 17. The Effects of Amine-carboxyboranes on 5'-Lipoxygenase Activity of IC-21 Cells After 5 Hours**

| | % of Control [N=6] | | |
|---|---|---|---|
| Concentration (μM) | COMPOUND #1 | COMPOUND #2 | COMPOUND #3 |
| 12.5 | 86±8** | 69±6* | 60±7* |
| 25 | 52±8* | 58±7* | 56±5* |
| 50 | 43±6* | 54±5* | 48±8* |

Control = 100±13 % (2902 DPM/mg protein);*p<0.0001;**p<0.025

**Table 18. The Effect of Amine-Carboxyboranes at 10 µM on Calcium Uptake into L929 Cells**

| | | % of Control [N=8] | | |
|---|---|---|---|---|
| Time (hours) | Control | COMPOUND #1 | COMPOUND #2 | COMPOUND #3 |
| 1.5 | 100±23[a] | 35±22* | 41±25* | 46±10* |
| 5 | 100±13[b] | 18±14* | 44±14* | 53±36* |
| 8 | 100±17[c] | 39±31* | 52±11* | 53±36* |
| 24 | 100±11[d] | 26±12* | 42±15* | 51±24* |
| 48 | 100±17[e] | 47±13* | 46±15* | 35±12* |

Control Values: [a] 2837 CPM/mg protein; [b] 5457 CPM/mg protein; [c] 3352 CPM/mg protein; [d] 3103 CPM/mg protein; [e] 2434 CPM/mg protein; * p<0.0001

---

The effects of pure recombinant human TNFα on calcium uptake in L929 cells can be compared to the effects of amine-carboxyboranes at 10 µM after 90 minutes. TNFα at 10 ng/ml and 100 ng/ml reduced calcium uptake into L929 cells by 33% and 40%, respectively as 90 min.[data not shown]. Pure recombinant human TNFα or amine-carboxyboranes were added to L929 cells and the effects on proline incorporation into collagen and non-collagen were compared for 48 hr. Proline incorporation into collagen was enhanced by the amine-carboxyboranes at 90 min. and at 24 hr. above control values.

At 8 hours, Compounds #1 and #3 caused a 32-34% reduction in proline incorporation into non-collagen of L929 cells. A moderate increase was observed at 90 min. Compound #2 was not effective in reducing proline incorporation into non-collagen of L929 cells at any time [Table 20]. TNFα at 10 or 100 ng/ml of medium did not affect the incorporation of proline into collagen of L929 cells; however, proline incorporation into non-collagen was reduced 24% at 10 ng but not at 100 ng/ml at 90 min. incubation.

---

**Table 19. The Effect of Amine-Carboxyboranes at 10 µM on Incorporation of Proline into Collagen in L929 Cells**

| | | % of Control [N=6] | | |
|---|---|---|---|---|
| Time (hours) | Control | COMPOUND #1 | COMPOUND #2 | COMPOUND #3 |
| 1.5 | 100±15[a] | 169±14* | 114±15 | 127±8* |
| 5 | 100±13[b] | 115±6 | 103±3 | 98±6 |
| 8 | 100±8[c] | 87±5 | 104±5 | 87±11 |
| 24 | 100±10[d] | 186±12* | 142±11* | 190±16* |
| 48 | 100±5[e] | 118±44 | 86±15 | 82±23 |

Control Values: [a] 3061 DPM/ml [d] 4738 DPM/ml *p<0.0001

[b] 2937 DPM/ml [e] 7084 DPM/ml [c] 2719 DPM/ml

---

Proline incorporation into collagen in L929 cells after 5 hours in the presence of amine-carboxyboarnes at 12.5, 25 and 50 µM was generally decreased (Table 21). Compound #1 caused a 31-33% reduction of incorporation of proline at all concentrations of drug and was the most effective agent. Compound #3 caused a 22-28% reduction at all concentrations and Compound #2 was not as effective producing a 14% reduction at 50 µM.

**Table 20. The Effect of Amine-Carboxyboranes at 10 μM on Proline Incorporation into Non-Collagen in L929 Cells**

| Time (hrs) | Control | COMPOUND #1 | COMPOUND #2 | COMPOUND #3 |
|---|---|---|---|---|
| | | % of Control[N=6] | | |
| 1.5 | 100±8[a] | 83±5 | 88±5 | 92±3 |
| 5 | 100±5[b] | 102±5 | 83±7 | 107±12 |
| 8 | 100±8[c] | 68±3* | 91±4 | 66±4* |
| 24 | 100±7[d] | 78±7* | 108±15 | 174±19* |
| 48 | 100±17[e] | 106±5 | 89±6 | 112±5 |

Control Values: [a] 285 DPM/ml; [b] 209 DPM/ml; [c] 310 DPM/ml; [d] 1519 DPM/ml; [e] 1266 DPM/ml; *p<0.0001.

---

Proline incorporation into non-collagen in L929 cells after 5 hours in the presence of amine-carboxyboranes at 12.5, 25 and 50 μM was not significantly changed at any concentration, but did decline 18% with Compound #3 at 50 μM. [Table 22].

---

**Table 21. The Effect of Amine-Carboxyboranes on Proline Incorporation into Collagen in L929 Cells After 5 Hours**

| Concentration (μM) | COMPOUND #1 | COMPOUND #2 | COMPOUND #3 |
|---|---|---|---|
| | % of Control(N=6) | | |
| 12.5 | 69±2* | 95±3 | 78±5* |
| 25 | 69±6* | 87±4 | 72±5* |
| 50 | 67±2* | 86±3 | 76±7* |

Control=100±5% (8780 DPM/ml);*p<0.0001.

---

**Table 22. The Effect of Amine-Carboxyboranes on Proline Incorporation into Non-Collagen in L929 Cells After 5 Hours**

| Concentration (μM) | COMPOUND #1 | COMPOUND #2 | COMPOUND #3 |
|---|---|---|---|
| | % of Control(N=6) | | |
| 12.5 | 111±3 | 95±11 | 100±5 |
| 25 | 87±10 | 92±11 | 100±5 |
| 50 | 103±10 | 111±15 | 82±13 |

Control=100±5% (800 DPM/ml).

---

In L929 cells, calcitonin high affinity binding to receptors was measured at 1.5, 5, 24, and 48 hours [Table 23]. High affinity calcitonin binding was decreased 22% by Compound #2 and 31% by Compound #3 at 5 hours. Compound #1 afforded the best reduction of calcitonin high affinity receptor binding at 48 hours (i.e. 30%) and the binding was actually elevated significantly above the control at 5 hours (i.e. 48%). High affinity binding of DHD in L929 cells at 90 minutes in the presence of amine-carboxyboranes at 10 μM demonstrated significant elevation, i.e. Compound #1 caused a 21% increase, Compound #2 a 54% increase and Compound #3 a 69% increase. Pure recombinant human TNFα added to L929 cells caused an increased DHD high affinity binding by 42% at 10 ng/ml and by 14% at 100 ng/ml.

**Discussion**

The amine-carboxyboranes appear to reduce TNFα binding in L929 fibroblasts from 30 to 120 min. The 10 μM concentration of the agents appeared to afford the best response in displacing the radioactive TNFα which suggest at this concentration the agents were competitive with the cytokine's binding to this L929 protein receptor. This receptor is important in the release of hydrolytic enzymes and proteolytic enzymes which initiate and cause tissue damage to tissue. The amine-

Table 23. The Effect of Amine-Carboxyboranes at 10 μM on High Affinity Binding of Calcitonin to Receptors on L929 Cells

% of Control (N=8)

| Time (hours) | Control | CMPD #1 | CMPD #2 | CMPD #3 |
|---|---|---|---|---|
| 1.5 | 100±15[a] | 104±12 | 82±6 | 77±8* |
| 5 | 100±13[b] | 148±15* | 78±14* | 69±8* |
| 24 | 100±8[c] | 117±11 | 66±13* | 78±8* |
| 48 | 100±10[d] | 70±13* | 151±20* | 143±13* |

Control Values: [a] 1526 CPM/mg protein; [b] 1207 CPM/mg protein; [c]1353CPM/mg protein; [d] 907CPM/mg protein; * p<0.0001

carboxyboranes have previously been shown to significantly reduce the release of these enzymes as well reduce their catalytic activities[1,3]. The present study demonstrated that the time frame for the reduction of acid phosphatase activity in the presence's of the agents is consistent with the time of maximum inhibition of TNFα high affinity receptor binding. The inhibition of TNFα receptor binding also correlated with the agents' ability to suppress the activity of the regulatory enzyme for prostaglandin synthesis at 90 min. Both the inhibition of hydrolytic enzyme activity and prostaglandin cyclo-oxygenase activity continued through 5 to 18 hours. Similar observation have been made when amine-carboxyboranes were incubated with bone UMR-106 cells in that these same biochemical parameters were first suppressed at 90 min. consistent with suppression of the TNFα receptor but continued for a much longer period of time than when peaked binding occurred with this cytokine receptor[7]. TNFα release was 120 ng/ml at 90 min the peak but was still at 80 ng/ml after 50 hr. well above background levels[7]. NAG hydrolytic activity was not inhibited until 48 hr. 5'-Lipoxygenase activity was suppressed after 5 hr rather than 90 min. The inhibition of this enzyme activity by the agents correlated more with the observed suppression IL-1 high affinity receptor binding than with suppression of TNFα high affinity binding. Similar parallels were observed when UMR-106 bone cells were examined for effects of amine-carboxyboranes on cytokine high affinity receptors[7]. Cytokines which are known to play a role in inflammation are TNFα, IL-1, Il-6 and IL-8. Apparently TNFα effects are early, e.g. 30 min to 6 hr., this is followed by Il-1 effects which begin around 2 hr and continue for 12 hr. IL-8 is released late and has its high affinity binding to its receptors from 24 to 48 hr.

Differences in response to the agents as well as the high affinity receptors for cytokines involved calcium uptake in L929 cells and bone UMR-106 cells Calcium uptake in bone cells peaked at 90 min and was apparently suppressed by TNFα or the agents binding to TNFα high affinity receptor at 90 min. but at 5 hr. this process was reversed and calcium uptake into bone cells was increased two fold at 5 to 8 hr[7]. In L929 cells calcium uptake peaked at 5 hr. but was suppressed by the agents from 90 min to 8 hr. The agents suppressed dihydrovitamin $D_3$ binding to its nuclear receptor early but in bone cells increased the binding at 8 to 24 hr. which correlates with the increased uptake calcium into bone cells. Calcitonin high affinity binding was not affected by the agents in a manner which would indicated it affects calcium uptake. In vivo the amine-carboxyboranes cause an elevation of PTH and 1,25-dihydrovitamin $D_3$ after 14 days administration which correlated directly with an increase in serum calcium levels[2]. Proline incorporation into collagen on the other hand. peaked in bone cells at 5 to 8 hr. while in L929 cells it peaked at 24 hr. in the presence of the agents. This process is required by both types of cells to increase bone tensile strength and to repair wounds. Thus, there does appear to be some tissue difference in response to the agents and the time of their maximum effects in a given type of cell.
Since the amine-carboxyboranes are very potent anti-inflammatory agents[1-4], the fact that they are able to block TNFα, IL-1 and IL-8 receptor binding acting as antagonists would certainly explain the ability of the agents to reduce the inflammation process. The early events of the inflammation process are probably mediated by invading macrophages and PMNs. These cells possess high affinity cytokine receptors which regulate the release and synthesis of hydrolytic and proteolytic enzymes which cause local tissue damage, vaodilation, generation of free radicals, infiltration of white blood cells, local heat, septic skock and anorexic wasting. The latter stage involves tissue repair conducted by fibroblasts which also possess high affinity receptors for cytokines. Both types of cells, macrophages and fibroblasts, contain high affinity receptors which regulate cellular events, their own release and the release of other cytokines[5,6]. This is a chain reaction over time; thus, it is important that an effective anti-inflammatory drug block sequel events in the reaction as well as stimulate the repair process. The amine-carboxyboranes appear to achieve these goals and further investigation as potential therapeutic agents is warranted.

**Reference**

1. I.H. Hall, K.G. Rajendran, S.Y. Chen, O.T. Wong, A. Sood, B.F. Spielvogel, Arch Pharm (Weinheim) **1995**, 328, 39-44.
2. K.G. Rajendran, S.Y. Chen, A. Sood, B.F. Spielvogel, I.H. Hall. Biomed. Pharmacotherapy. **1995**, in press.
3. I.H. Hall, S.Y. Chen, K.G Rajendran, A. Sood, B.F. Spielvogel, J. Shih, Environ. Health Perspect **1994**, 102 (Suppl 3), 21-30.
4. I.H. Hall, C.O. Starnes, A.T. McPhail, P. Wisian-Neilson, M.K. Das, B.F. Spielvogel, J. Pharm. Sci.**1980**, 69, 1025-1029.
5. C.J. Dunn, "Cytokines as mediators of chronic disease" in Cytokine and Inflammation, ed. E.S. Kimball, CRC Press, Boca Raton, Fl. **1991**, 1-33.
6. C.A. Dinarello, "Role of interleukin-1 and tumor necrosis factor in systemic response to infection and inflammation" in Inflammation: Basic Principles and Clinical Correlates, ed. J.I. Gallin, Raven Press, Ltd. , N.Y., **1992**, 211-232.

7. M.E. Murphy, A.I. Elkins, R.P. Shrewsbury, A. Sood, B.F. Spielvogel, and I.H. Hall, Arch Pharm. submitted.
8. F.C. Kull, Jr., Nat Immun. Cell Growth Regul. **1988**, 7, 254-265.
9. F.H. Nielsen, C.D. Hunt, L.M. Mullen, J.R. Hunt, FASEB J., **1987**, 1, 394-397.
10. F.H. Nielsen, L.M. Mullen, S.K. Gallagher, J. Trace Elements Exp. Med. **1990**, 3, 45-54.
11. B. Peterofsky, Arch Biochem. Biophys. **1972**, 152, 318-325.
12. P.S. Chen, T.Y. Toribara, and H. Warner,. Anal. Chem.**1956**, *28*, 1756-1758.
13. H. Tulberg-Reinert, A.F.Hefti, Agents and Actions **1977**, 32, 321-332.
14. R.V. Tomlinson, R.V. Ringold, M.C. Qureshi, E. Forchieli, Biochem. Biophys. Res. Commun. **1972**, *46, 552-558*..
15 M. Glatt, H. Kalin, K. Wagner, K. Brune, Agents Actions **1977**, 7, 321-334.
16. D.L. Flynn, T.R. Belliotti, A.M. Boctor, D.T. Connor, C.R. Kostlan, D.E.,Nies, D.F. Ortwine, D.J. Schrier, J.C. Sircar, J. Med. Chem. **1991**, 34, 518-525.
17. D.L. Flynn, T.Capiris, W.J. Cetenko, D.T. Connor, R.D. Dyer, C.R. Kostlan, D.E. Nies, D.J. Schrier, J.C. Sirar, J. Med. Chem., **1990**, *33*, 2070-2072.
18. I.H. Hall, R. Simlot, C. Oswald, A.R.K. Murthy, H. ElSourady, J. Chapman Jr., Acta Pharm. Nord. **1990**, 2, 387-400.

**Received: July 14, 1995 - Accepted: August 23, 1997 - Accepted in revised camera-ready format: September 12, 1995**

